# Telomere shortening in breast cancer cells (MCF7) under treatment with low doses of the benzylisoquinoline alkaloid chelidonine

**DOI:** 10.1371/journal.pone.0204901

**Published:** 2018-10-03

**Authors:** Sakineh Kazemi Noureini, Leili Fatemi, Michael Wink

**Affiliations:** 1 Department of Biology, Faculty of Basic Sciences, Hakim Sabzevari University, Sabzevar, Iran; 2 Department of Biology, Institute of Pharmacy and Molecular Biotechnology, Heidelberg University, Heidelberg, Germany; Tulane University Health Sciences Center, UNITED STATES

## Abstract

Telomeres, the specialized dynamic structures at chromosome ends, regularly shrink with every replication. Thus, they function as an internal molecular clock counting down the number of cell divisions. However, most cancer cells escape this limitation by activating telomerase, which can maintain telomere length. Previous studies showed that the benzylisoquinoline alkaloid chelidonine stimulates multiple modes of cell death and strongly down-regulates telomerase. It is still unknown if down-regulation of telomerase by chelidonine boosts substantial telomere shortening. The breast cancer cell line MCF7 was sequentially treated with very low concentrations of chelidonine over several cell passages. Telomere length and telomerase activity were measured by a monochrome multiplex quantitative PCR and a q-TRAP assay, respectively. Changes in population size and doubling time correlated well with telomerase inhibition and telomere shortening. MCF7 cell growth was arrested completely after three sequential treatments with 0.1 μM chelidonine, each ending after 48 h, while telomere length was reduced to almost 10% of the untreated control. However, treatment with 0.01 μM chelidonine did not have any apparent consequence. In addition to dose and time dependent telomerase inhibition, chelidonine changed the splicing pattern of hTERT towards non-enzyme coding isoforms of the transcript. In conclusion, telomere length and telomere stability are strongly affected by chelidonine in addition to microtubule formation.

## Introduction

Telomeres are specialized nucleoprotein structures at the ends of linear eukaryotic chromosomes which were first observed in 1938 by Muller [1,2]. Their function is essential for the stability and protection of chromosomes from degradation by DNases [2,3], preventing end-joining [3] and aberrant recombination of chromosomes [2,4]. In humans, telomeres with a length of approximately 5–15 kb are composed of tandem repeat of a noncoding sequence of 5'-TTAGGG-3' and associated proteins TRF1, TRF2, RAP1, TPP1, POT1, TIN2 that constitute the so-called shelterin complex [5–8]. When telomeres are long enough, chromosomes work properly in cells. However, in cycling cells, telomere shortening because of the end-replication problem leads to reduction of telomere length by 50–100 base pairs after every cell division [1,2,9–11]. Therefore, telomeres play critical roles as a molecular clock which determines the number of cellular divisions [12,13]. Critically short telomeres activate intracellular signalling pathways which can induce cell cycle arrest and programmed cell death [14,15].

Telomerase is a ribonucleoprotein enzyme with reverse transcriptase activity that extends 3´ termini of DNA strand by adding TTAGGG repeats [16, 17]. Telomerase is active in about 90% of cancers but not in normal somatic cells. Therefore, telomerase and telomeres have been targeted for cancer treatment [18, 19]. Although telomerase is critical for telomere length maintenance in cancer cells, the telomere length in chemotherapeutically treated cells may be independent of telomerase activity by using an alternative mechanism involving non-homologous end joining at telomeres (see reference [20] for review).

*Chelidonium majus* (family Papaveraceae) produces several valuable alkaloids. Various pharmacological actions such as antiviral, anticancer, antibacterial/antifungal, and anti-inflammatory effects have been reported for this plant [21–23]. A recent study also reported novel insecticidal and larvicidal effects of this plant [24] Chelidonine, the most abundant benzophenanthridine alkaloid in *C*. *majus*, blocks cell growth in several cell types and its effectiveness has been investigated in mice with diverse tumours (see [21] for review). Chelidonine is also well known for its strong anti-proliferative properties in cancer cells through inhibition of tubulin polymerization [25,26]. Our previous screening studies showed a strong inhibitory effect of this alkaloid on telomerase and stimulation of cell death in HepG2 [27] and MCF7 cells [28]. The treated cells morphologically appeared to be aged with a large cell volume and high cytoplasmic to nuclear ratio. Induction of senescence in long-term treated cells has been demonstrated using β-galactosidase activity, a commonly used biomarker for cell senescence [27,28]. However, considerable fractions of the cell population follow other pathways including apoptosis and autophagy [27,28]. Investigating mechanisms involved in anti-telomerase activities of the isoquinoline alkaloids of *C*. *majus*, we observed various levels of interaction with telomeric sequences. Chelidonine, although repressing the enzyme activity through hTERT gene down-regulation, has almost no significant interaction with telomeric oligonucleotide [29]. However, the actual effect of chelidonine on telomeres as the main chromosomal targets inside the cells is still to be investigated.

Here, we determined telomere length of MCF7 breast cancer cells under long term treatment with low concentrations of chelidonine using a very sensitive and improved monochrome multiplex quantitative PCR (MMQPCR) method.

## Materials and methods

### Cytotoxicity

Human breast adenocarcinoma cell line MCF7 (ACC115 from DSMZ) was maintained in 75 cm^2^ culture flasks in DMEM, supplemented with 10% heat-inactivated fetal bovine serum, 100 U/ml penicillin, and 100 μg/ml streptomycin (all the materials from PAA, Austria). Chelidonine (≥97% HPLC) was purchased from Sigma-Aldrich. Cells were grown in 5% CO_2_ and 95% air atmosphere at 37°C. Cell viability was evaluated routinely by the trypan blue exclusion method using a hemocytometer. Cytotoxicity of chelidonine was determined using the MTT test [30]. Briefly, exponentially growing cells were seeded in 96 well plates, 10000 cells per well, and after 24 h incubated in medium containing various concentrations of chelidonine freshly prepared by dilutions (100, 50, 25, 12.5, 6.25, 3.12, 1.56, 0.78, 0.39, 0.2 and 0.02 μM) from a stock solution (50 mM in absolute ethanol), while the final concentration of ethanol was always less than 0.01%. After 48 h incubation, MTT (3-(4,5-dimethylthiazol-2-yl)-2,5-diphenyl-tetrazolium bromide, Sigma-Aldrich) was added to a final concentration of 0.5 mg/ml to each well. Incubation was continued for four hours for MTT reduction to the purple formazan product by mitochondrial dehydrogenases of viable cells and after dissolving it in dimethylsulfoxide including 10% SDS and 1% acetic acid, the absorbance was measured at 570 nm using a plate reader (BioTek, USA). The analyses were repeated four times each in triplicate and the 50% lethal dosage, LD_50_ values were calculated from dose-response curves using Gen5 version 1.06 software.

### Cell culture

The MCF7 cell line was maintained in 5% CO_2_ at 37°C and 95% humidity in DMEM High glucose with glutamine (PAA, Austria) culture medium, supplemented with 10% heat-inactivated FBS. Viability of the cells was evaluated with trypan blue staining. For telomere length measurements, the cells were seeded in 25 cm^3^ culture flasks. On the next day they were treated with 0.01, 0.05 or 0.1 μM chelidonine in fresh medium for 48 h, after which the corresponding medium was substituted with normal medium. After harvesting and counting the cells, 3 × 10^5^ cells were sub-cultured and DNA extraction was done on the rest. This procedure was continued as long as the required number of cells was available for plating. For this reason, serial treatments of the cells with 0.1 μM chelidonine could not be continued after the fourth confluence.

In a different experiment cells were grown and treated as above, while chelidonine treatments, 48 h per passage, were stopped at the desired time: after one, two, three or four sequential subcultures. Cell viability was monitored thereafter while normal medium was substituted. In each subculture cell viability was evaluated using MTT method as mentioned above. The experiment was repeated three times using triplicated samples for each concentration.

### DNA extraction

Genomic DNA was extracted from treated and control cell samples collected from every passage. Briefly, cell pellets were incubated overnight at 55°C in 500 μl of lysis buffer containing 0.05 M Tris-HCl pH = 8.0, 0.05 M EDTA, 0.5 M NaCl, 10% sodium dodecyl sulphate, 0.05 M DTT, 5 μl of proteinase K, followed by NaCl extraction, isopropanol and ethanol precipitation, and stored at -20°C. The extracted DNA samples were checked for purity and quality by agarose gel electrophoresis. The DNA concentrations were determined by NanoDrop 1000.

### Telomere length assay

The relative average telomere length of the DNA samples was determined using the monochrome multiplex quantitative PCR (MMQPCR) method in a Rotor-Gene 3000 real-time System [31,32].

In brief, the reagents in the 25 μl PCR were 10 mM Tris-HCl (pH 8.3), 3 mM MgCl_2_, 63 mM KCl, 1 mM EGTA, 0.005% Tween 20, 0.1 mg/ml BSA, 0.2 mM each of dNTP, 1 M betaine, 0.75 × SYBR Green I, 0.625 U AmpliTaq Gold DNA polymerase and 300 ng of genomic DNA. The four primers (at 900 nM) were (5′ to 3′): telg, ACACTAAGGTTTGGGTTTGGGTTTGGGTTTGGGTTAGTGT; telc, TGTTAGGTATCCCTATCCCTATCCCTATCCCTATCCCTAACA; albu: CGGCGGCGGGCGGCGCGGGCTGGGCGGaaatgctgcacagaatccttg; albd: GCCCGGCCCGCCGCGCCCGTCCCGCCGgaaaagcatggtcgcctgtt.

Real time PCR was performed following Cawthon (2009), using the following cycling profiles: for telomere amplification, 1 cycle of 10 min at 94°C; 2 cycles of 2 s at 98°C, 15 s at 49°C; 40 cycles of 2 s at 98°C, 10 s at 64.5°C, 15 s at 72°C with signal acquisition, 10 s at 84°C, and 15 s at 88°C with signal acquisition. The 74°C reads provided the Ct for telomeres; the 88°C reads provided the Ct for the single copy gene (albumin). Five serially diluted samples of the reference DNA (extracted from untreated control MCF7 cells) spanning 3–300 ng were included for standard curves. Two standard curves were generated by the rotor-gene 6_1_93 software for telomere and albumin. Each experimental or standard sample was assayed in duplicate and the average amounts were reported.

Relative average telomere lengths are expressed as the T/S ratio. For a given experimental sample, the T value is the amount of the reference DNA (in ng) that matches the experimental sample for copy number of the telomere template, and the S value is the amount of the reference DNA (in ng) that matches the experimental sample for copy number of the single copy gene template. The single copy gene used as a reference was albumin. Samples with a T/S > 1.0 have an average telomere length greater than that of the standard DNA; samples with a T/S < 1.0 have an average telomere length shorter than that of the standard DNA.

### Population doubling time

Population doublings and the doubling time, the time required for a cell culture to double in number, were calculated as PD = (log_10_n_f_ −log_10_n_i_)/0.301 and DT = log_10_2T/log_10_n_f_ −log_10_n_i_ respectively, where n_i_ is the initial number of cells and n_f_ is the final number of cells at each passage and T is time in hours.

### q-TRAP assay

MCF7 cells were seeded in six well plates with 10^5^ cells per well and after 24, 48 and 72 h incubation with various concentrations of chelidonine (0.1, 1 and 8 μM) they were trypsinized and washed with cold PBS. Cells were incubated in lysis buffer containing 10 mM Tris–HCl pH = 7.5, 1 mM MgCl_2_, 1 mM EGTA, 0.1 mM phenylmethylsulfonylfluoride (PMSF), 5 mM beta-mercaptoethanol, 0.5% CHAPS and 10% glycerol for 30 min [27]. The supernatant was collected after 30 min centrifugation at 14,000 x *g* and the protein concentration was determined using the Bradford assay.

The total volume of the q-TRAP reaction mixture was 20 μL and contained 10 μl SYBR Green Kit, 10 pM primer TS 5´-AATCCGTCGAGCAGAGTT-3´ and H_2_O (DEPC). The reaction mixture was incubated at 25°C for 20 min. Then, after adding 5 pM ACX 5´-GCGCGG(CTTACC)_3_CTAACC-3´, real-time PCR was performed using the cycling profile: 1 cycle of 10 min at 95°C; 40 cycles of 30 s at 94°C, 30 s at 50°C, 45 s at 72°C [33].

### Total RNA isolation, cDNA synthesis and estimation of alternative hTERT splicing variants

Total RNA was isolated from the control and the 48 h treated MCF-7 cells using RNX-Plus solution (SinaClone BioScience, Iran) according to the manufacturer’s instructions, and cDNA synthesis was performed with 2 μg of each sample using MMULV reverse transcriptase (Vivantis, Korea). The expression level of hTERT and β2 microglobulin genes was determined using the quantitative reverse transcription polymerase chain reaction (qRT-PCR) with specific intron-spanning primers as explained above [28]. Briefly, 1 μl of each cDNA sample was subjected to the PCR-amplification reaction including SYBR Green PCR Master Mix (GenetBio, Korea) and 5 pmol of each primer. The ratio of mRNA copy number of hTERT gene to the housekeeping gene β2 microglobulin was compared between the control and treated samples using the related standard curves calculated by Rotor-Gene 6.01 software. The hTERT transcript that encodes the catalytic subunit of telomerase has at least six alternate splicing sites including four insertions in human cancer cells [34, 35], which cause premature translation terminations. To examine the likely effects of the alkaloids on the splicing pattern of hTERT, the functional full length and defective transcripts were amplified using specific primers (hTERT-2026F: GCCTGAGCTGTACTTTGTCAA and hTERT-2482R: CGCAAACAGCTTGTTCTCCATGTC) for its exon junction spanning the RT region of hTERT [36]. This region contains α and β splicing sites and products of four different sizes may potentially be seen. The hTERT mRNA with a full length transcript is identified as hTERT +α+β (457 bp). This transcript is translated to the functional hTERT. Three other products do not encode the functional enzyme and are identified as hTERT-α (α variant; 421 bp), the major isoform hTERT-β (β variant; 275 bp), and hTERT-α-β (-α-β variant; 239 bp).

### Statistical analysis

Statistical analysis was performed using one-way ANOVA followed by Tukey’s HSD test and a *p* < 0.05 was considered as the cut off for significant differences.

## Results and discussion

### Chelidonine exhibited dose dependent cytotoxicity

**Fig 1:**
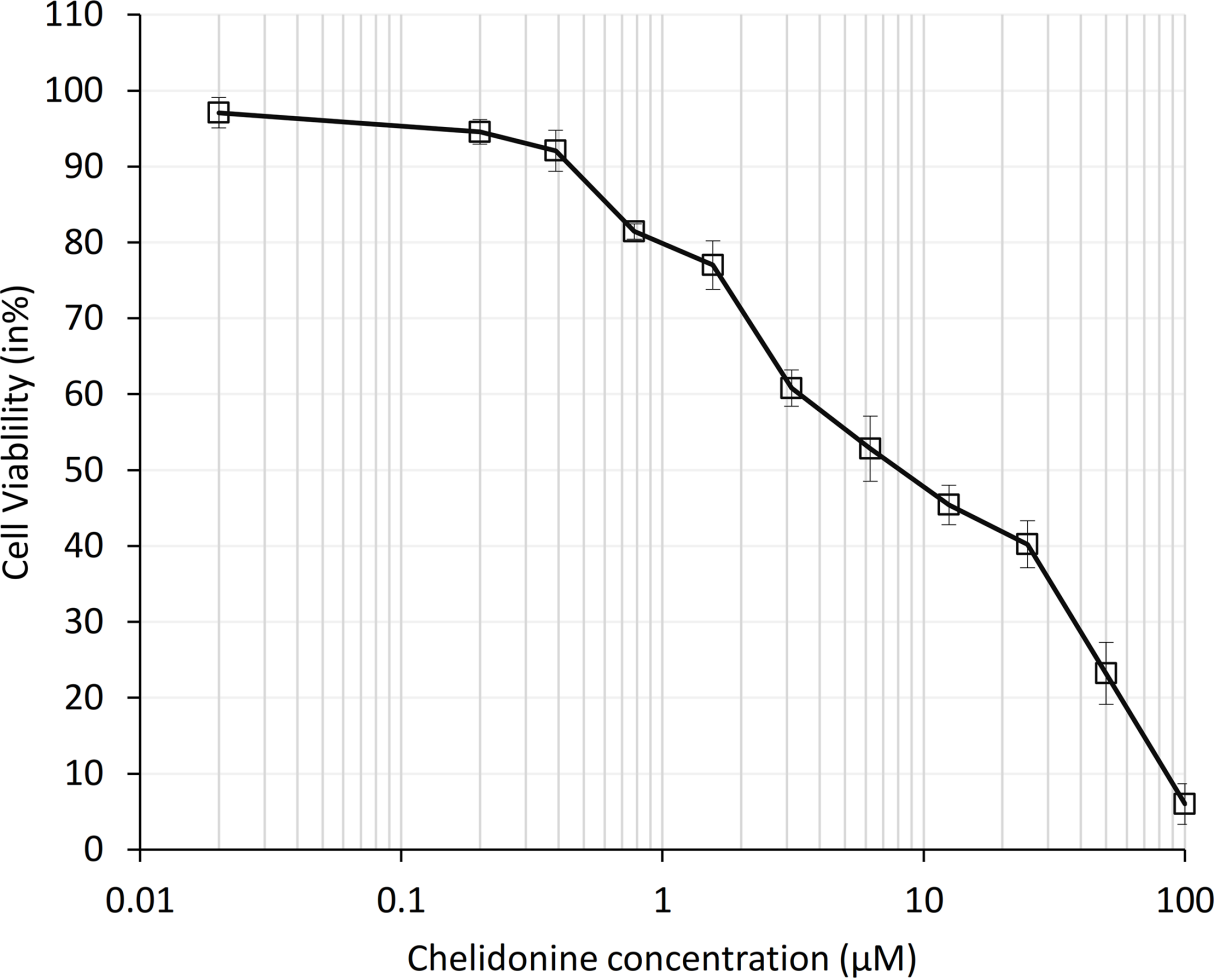
Cell viability of MCF7 cells after 48 h treatment with different concentrations of chelidonine was estimated using MTT test; mean values of four independent experiments ± SEM are shown.

The MTT method was used to assess the cytotoxicity of chelidonine in MCF7 cells. The LD_50_ value was 8 μM after 48 h treatment (p≤0.05). Chelidonine showed strong cytotoxicity, rapidly reducing viable cell numbers at low concentrations (Fig 1). However, this steep slope in the dose-response curve was subsequently moderated so that 20–30% of cells were still viable at 50 μM. A complete cell death was seen at 100 μM. In the following experiments very low concentrations: 0.01 and 0.05 μM, were used in long term treatments. In telomere length studies treatment with 0.1 μM chelidonine was included too.

### Chelidonine increased population doubling time

**Fig 2:**
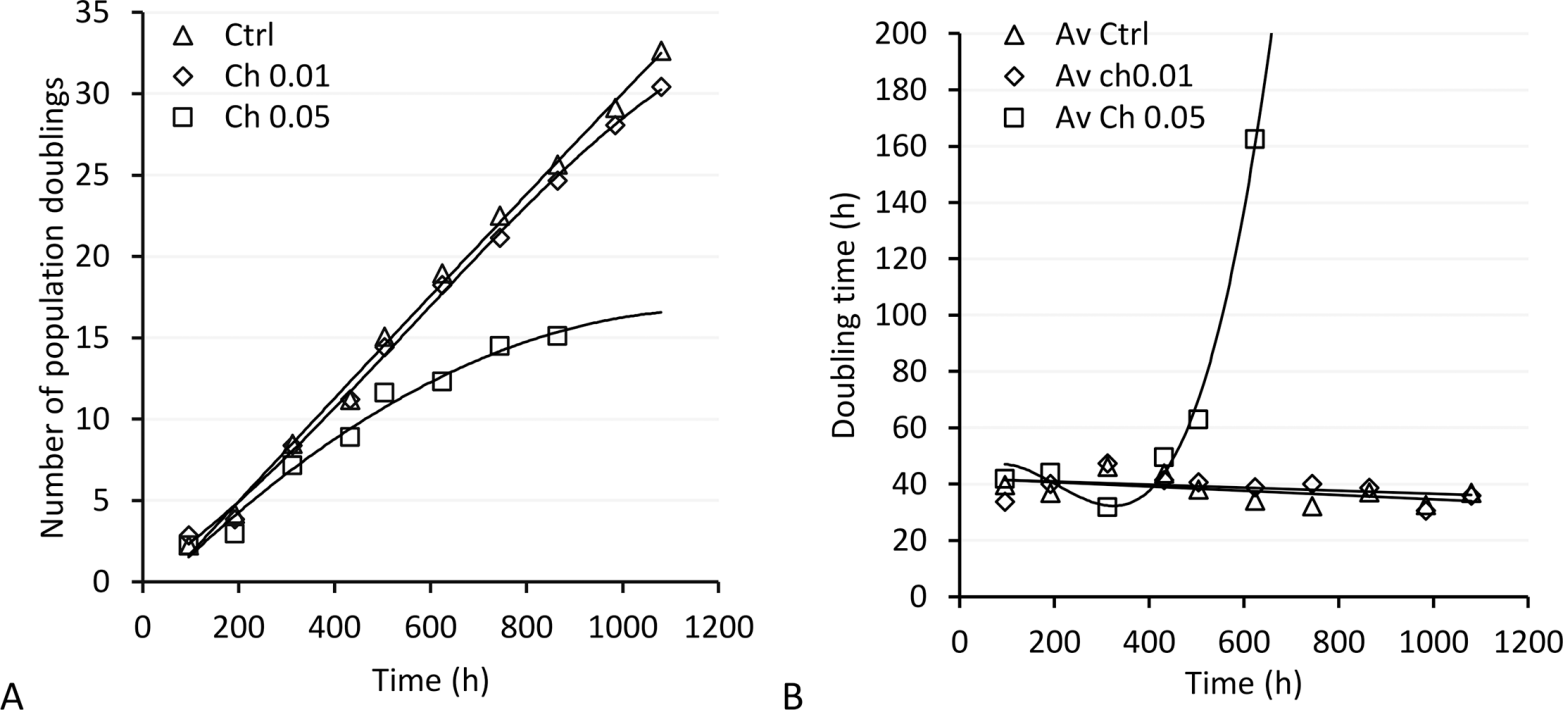
A) Number of population doublings and B) doubling time after long-term treatment with chelidonine (0.01; diamonds or 0.05 μM; squares) in comparison with untreated control MCF7 cells (triangles).

MCF7 cells were treated with 0.01 or 0.05 μM chelidonine for 48 h after each passage. Chelidonine at 0.01 μM did not change population doublings and doubling time of MCF7 cells significantly; no morphological change towards senescence or alteration of growth rates was observed even after continuous treatments of log-phase cultures for almost 1080 h (Fig 2, diamonds). However, a significant reduction of the growth rate occurred in cells treated with 0.05 μM chelidonine in comparison with untreated control (*p* < 0.005) which is clearly seen after five treatments (Fig 2, squares). At this time point, the treated cells showed approximately 30% less doublings in comparison with the control group. Doubling period in the treated cells was 162.5 ± 0.5 h as compared with 32.6 ± 0.5 h in control cells.

### Chelidonine strongly reduced telomere length in MCF7

**Fig 3:**
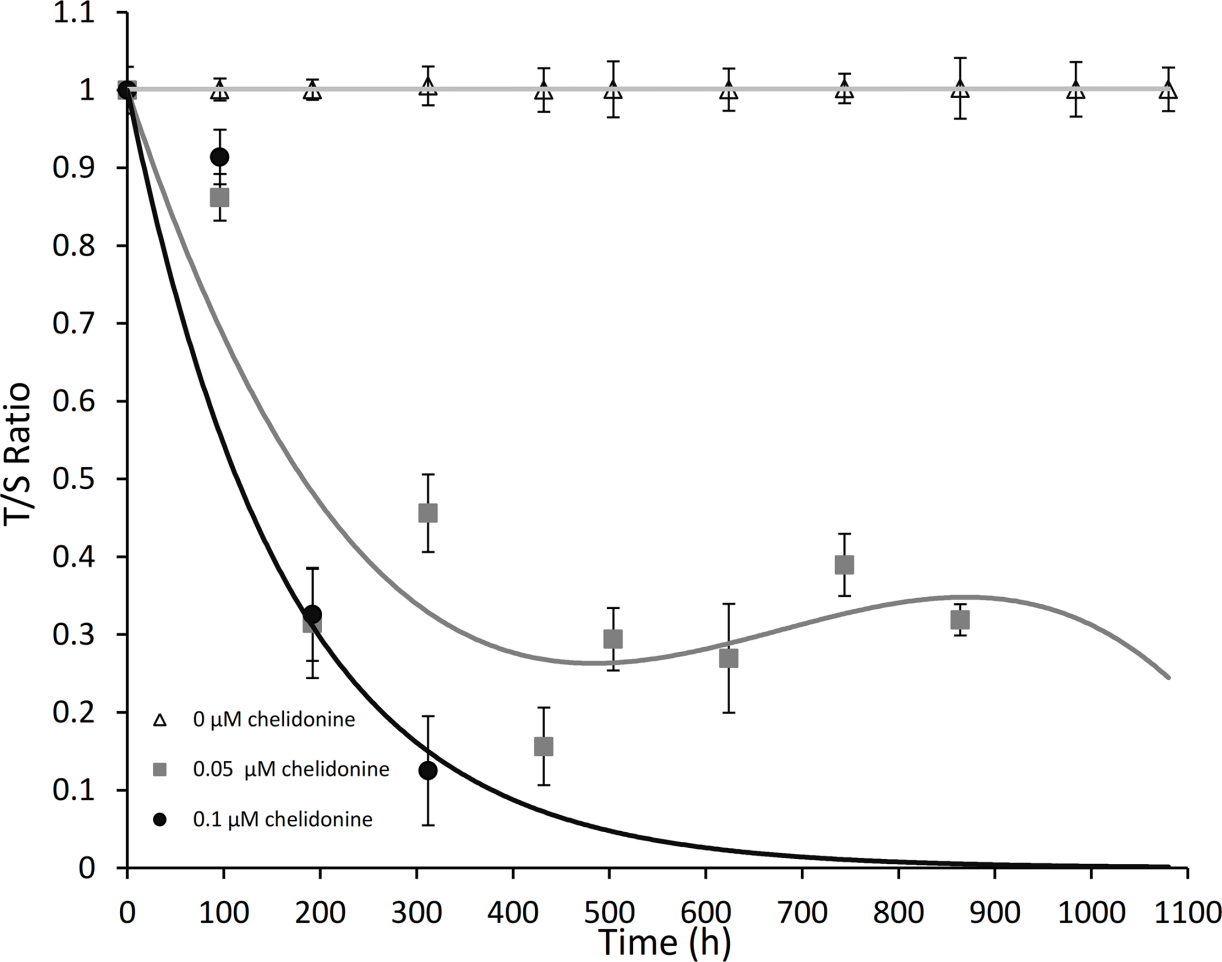
T/S values of cells sequentially treated with 0.05 μM (grey) and 0.1 μM (black) chelidonine 48 h in every passage were compared with telomere length of un-treated MCF7 cells (white) which was considered as 1. Data represented are the average of two independent experiments each in duplicates ± SD values.

Relative average telomere length can be measured by MMQPCR using primers that hybridize with the telomere hexamer repeats because the number of binding sites for the primers increases as average telomere length increases [37,38]. However, MMQPCR simultaneously counts the copy number of albumin as a single copy gene in each reaction tube using a specific primer pair, so the relative amount of telomere copies (T) to albumin (S) of the untreated control is measured. A representative experiment for the potential effects of chelidonine on MCF7 cells is shown in Fig 3. The T/S value of the treated samples in sequential treatments, 48 h per passage, was compared with that of un-treated cells, which was considered as 1 (triangles). The cells treated with 0.01 μM chelidonine showed only a minor decrease in telomere length after several treatments; as the ratio was not stably lower than 1. However, a diminished T/S ratio was observed soon after treatment with 0.05 μM chelidonine (squares). The T/S ratio decrease continued to less than 0.3 after 5 sequential treatments, implying telomere shortening to about 30% of the un-treated control cells. A rapid telomere loss was observed at 0.1 μM chelidonine (circles), so that plating became impossible after only three sequential treatments.

### Chelidonine strongly suppresses telomerase activity and hTERT transcription

**Fig 4:**
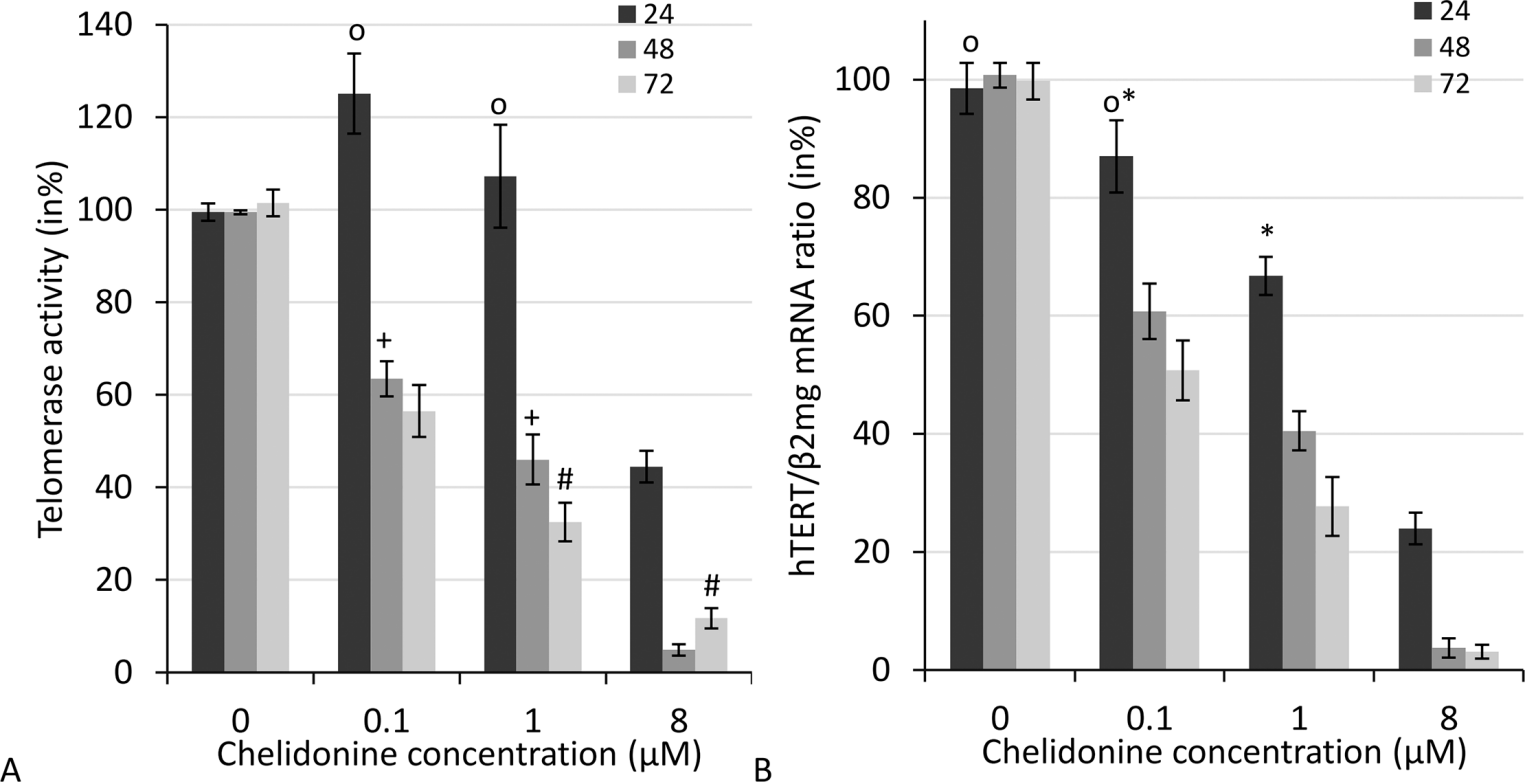
A) Telomerase activity as measured by q-TRAP assay and B) hTERT transcription levels using quantitative real-time RT-PCR technique in MCF-7 cells after various time of treatment with different concentrations of chelidonine. Mean value ±SEM of three logically independent experiments each containing three samples for each point was presented. (p≤0.01 in Pair-Wise Comparisons via Tukey HSD Test unless marked as + /# /* in the plot evaluating p ≤0.05. “o “represents no significance).

Quantitative telomerase repeat amplification protocol (qTRAP) measurements showed considerable reduction of telomerase activity in treated MCF7 cells. In other words, chelidonine reduces active telomerase time- and dose-dependently. Fig 4A shows the relative telomerase activity of MCF7 cells after 24, 48 and 72 h treatments with various concentrations of chelidonine. Telomerase activity was reduced ≥40% after 48 h treatment with 0.1 μM chelidonine. The IC_50_ value for telomerase inhibition in treated cells at this time point was 0.45 ± 0.08 μM (P≤0.05). The method measures the functional amount of the enzyme in equal amounts of total protein. Chelidonine also showed a robust suppression of hTERT transcription which was both time- and concentration-dependent (Fig 4B). The decreased enzyme activity and hTERT mRNA level after 48 h show almost the same pattern, while in a shorter time, 24 h, transcription decrease precedes the loss of enzyme activity.

### Chelidonine suppresses cell growth reversibly

**Fig 5:**
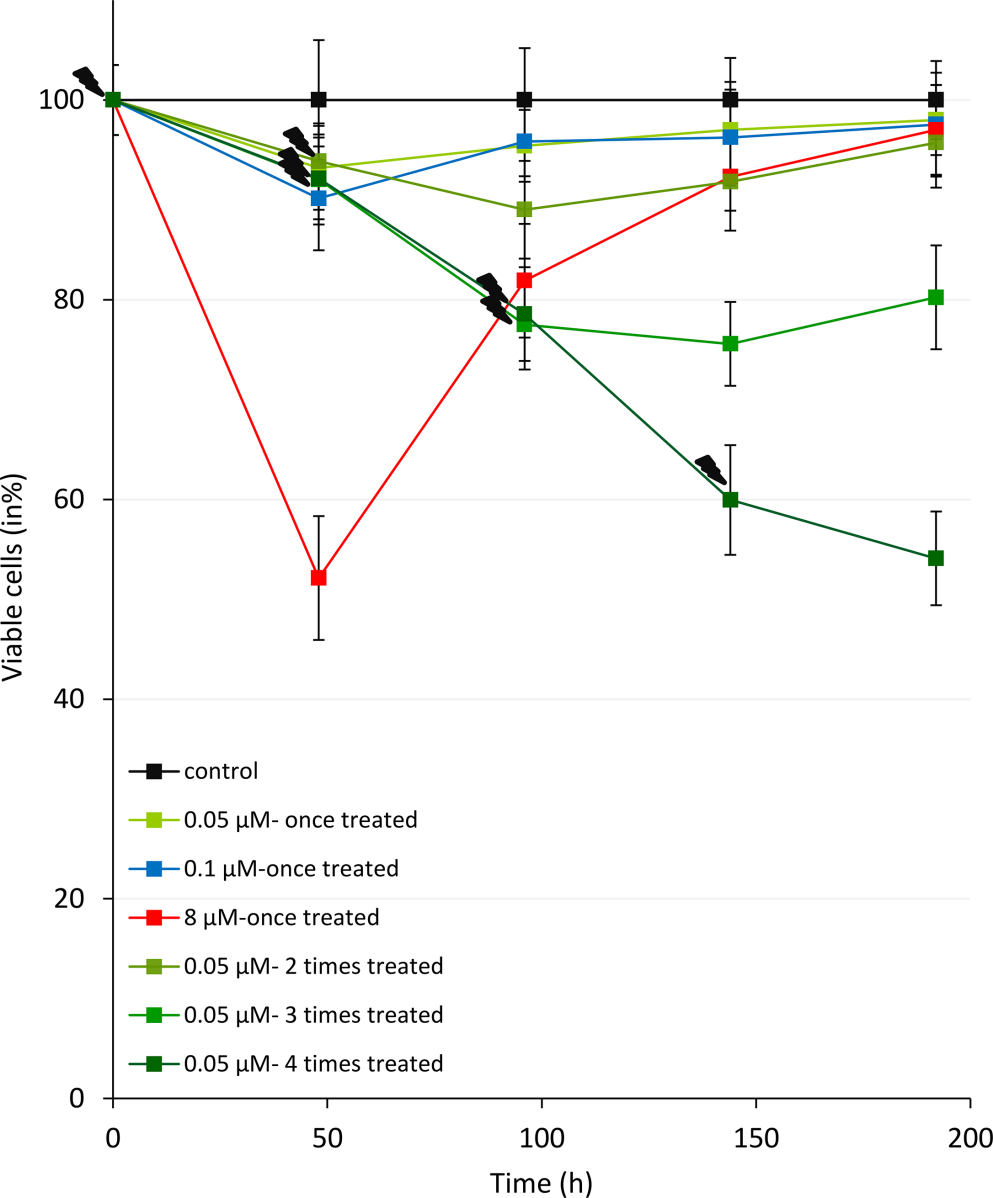
Cell viability of MCF7 cells after treatments (depicted with lightning bolts) 48h per passage with various concentration of chelidonine evaluated using MTT method (various concentrations and times of treatments were depicted using colour index in the chart). The mean ± SD values of nine samples were presented.

MCF7 cells that treated once for 48 h with 0.1 or 8 μM chelidonine retained some of their viability/growth rate when compared with un-treated control cells (Fig 5). Also, the cells treated sequentially 2 or 3 times with 0.05 μM chelidonine recovered their growth rate after the compound was removed from the medium. However, after four times treatment, the cell growth was so slow that replating was impossible. As seen in Fig 5 this shows that growth inhibition by chelidonine is reversible; cells mostly recovered their growth after removing chelidonine as far was observed.

### Chelidonine shifts the splicing pattern of hTERT towards non-enzyme coding variants

**Fig 6:**
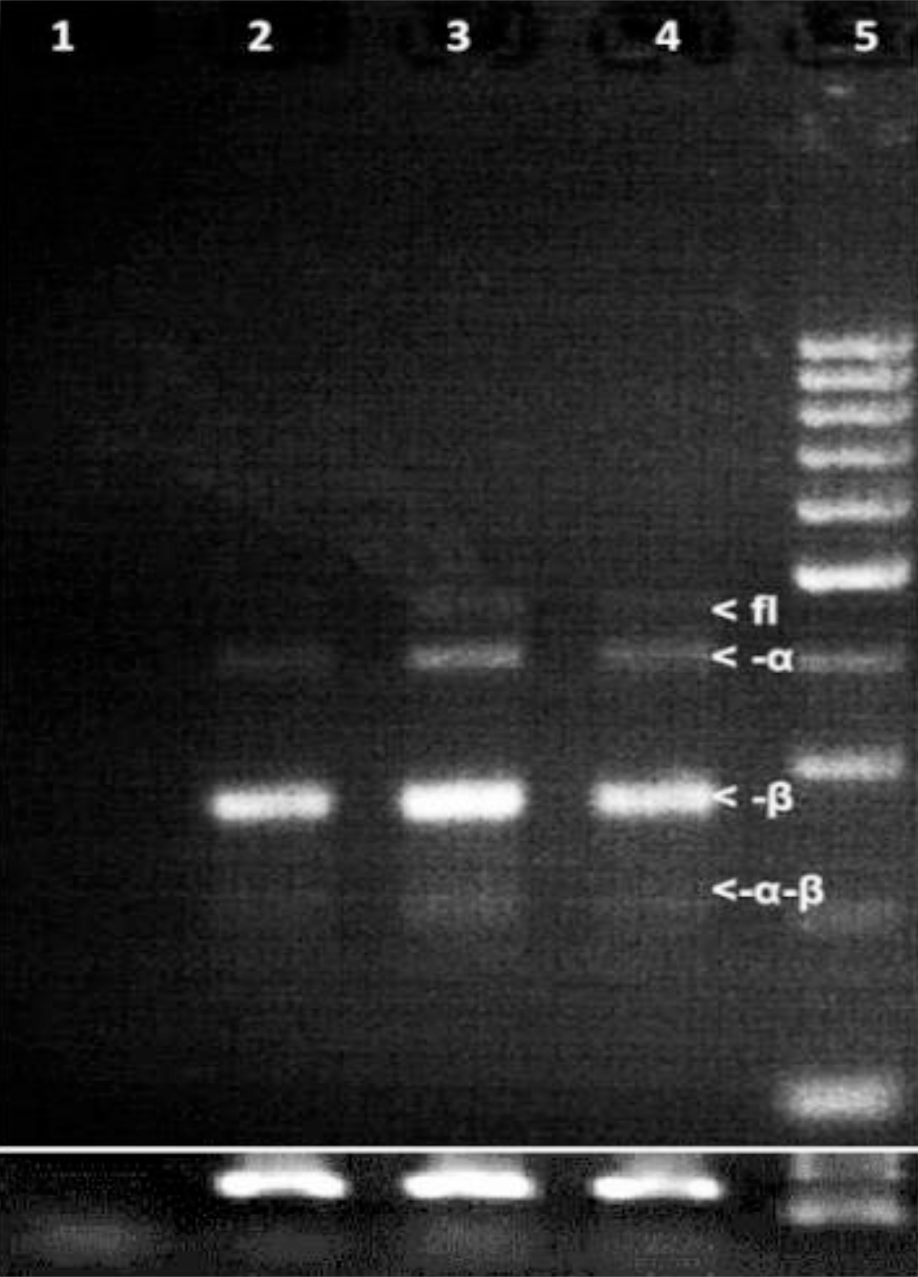
Alternative splice variants of hTERT from MCF-7 cells after 48 h treatment with 2 and 8 μM chelidonine in parallel with that of untreated cells. PCR products were analysed by gel electrophoresis (3% agarose gels). The white arrows show the location of four splice variants in untreated cells. The upper band is the functional full-length hTERT (FL, 457 bp) which is followed by the three shorter non-enzyme coding variants. Lane 1: negative control, 2: treatment with 5 μM chelidonine, 3: treatment with 2 μM chelidonine, 4: untreated control and 5: 100 bp DNA marker from which 10, 10, 10, 5 and 5 μl was loaded respectively; as seen in Fig 4B the total transcription of hTERT was strongly repressed while the major isoform is minus beta. Five microliters of β2-microglobulin PCR products of the related samples have been loaded as control at bottom.

Chelidonine reduced the total transcription level of hTERT dose dependently, as seen in Fig 4. The primers used cannot differentiate between the variants. However, telomerase, which is mainly regulated at the transcription level, has non-enzyme coding transcripts too. Transcripts of hTERT undergo a special alternative splicing in MCF-7, which results in at least four different splice variants. Among them the –β variant always figures as the most abundant form while the full length variant is the only active one. Chelidonine induced a clear shift in the splicing pattern of hTERT transcripts, although only at relatively high concentrations (Fig 6). The full-length transcript almost disappeared at the LD_50_ value, while it is still visible with 2 μM chelidonine treating. This implies that hTERT splicing is not considerably affected by low concentrations of chelidonine. High concentrations, however, suppress both total transcription and full length active variant of hTERT.

## Discussion

Telomere shortening normally occurs when cells are cycling. Short telomeres eventually lead to genomic instability and cell death. Short telomeres activate p53 and ATM/ATR kinases that prevent tumorigenesis [39]. Most cancer cells up-regulate telomerase activity necessary to maintain telomere length although this is shorter than in embryonic cells. Thus, both telomeres and telomerase play critical roles in developing cancer and both could be targets for anticancer medications [40]. However, telomerase inhibition is not always accompanied by telomere shortening, as telomere length reduction may be compensated by stimulation of non-homologous end joining NHEJ, which acts through recombination mechanisms [9]. Here we investigated telomere length in tumour cells treated with chelidonine for repeated cell cycles.

Chelidonine showed a potent time- and dose-dependent inhibition of telomerase transcription and translation. However, telomere length assays using the monochrome multiplex quantitative PCR method in MCF7 breast cancer cells showed a stably progressive decrease of telomere length when cells were repetitively treated with 0.1 μM chelidonine. Cell growth was completely arrested after the third treatment. A relatively mild response was observed with 0.05 μM chelidonine: telomere length had decreased to about 30% of the un-treated sample control after 5 passages. However, chelidonine did not show a stable and apparent telomere shortening at a concentration of 0.01 μM even after 10 serial passages, which may be inferred to mean reversible and/or transient inhibition at very low doses. It is noteworthy that telomere length regulation is a multifactorial process which is under precise control. Therefore, chelidonine at 0.01 μM may fail to complete destruction of the strongly regulated telomere interactome. An alternative mechanism of telomere protection exists involving non-homologous end joining (NHEJ) at telomeres. Whether the very low concentrations of chelidonine can stimulate NHEJ or not requires further investigation. However, in our previous report, HepG2 cells also showed growth arrest and senescence induction after a few treatments with low doses of chelidonine. Both HepG2 and MCF7 cells have relatively short telomeres: 6.1 kb [41] and 5.2 kb—6±1 kb [42,43], respectively, based on reports using TRF analysis. Therefore, this effect probably depends on the initial length of telomeres and needs further analysis.

Cell viability and proliferation of the cells were checked while sequentially treated. Fig 5 showed a remarkable reversibility in growth inhibition by chelidonine. The cells after one, two and three times treatments have less viability, yet recover their proliferation slowly after removing chelidonine. Data presented in Fig 3 indicates short telomeres after sequential treatments with 0.05 μM chelidonine; telomeres get very short (T/S ratio to about 0.3 of untreated cells after a few treatments). Comparison between the telomere length and cell viability data (presented in Figs 3 and 5 respectively) might lead to the inference that proliferation is blocked by an incomplete reduction of telomere length, although it is most likely reversible. Chelidonine is known to affect cell viability of MCF7 by stimulating various outcomes including apoptosis, autophagy and senescence in a dose dependent manner [29]. At 0.05 μM, chelidonine induces cell senescence in MCF7 cells [29]. Some small population of the treated cells may probably revive and proliferate slowly, although this is much weaker in sequential treating.

Chelidonine, which is known to inhibit mitosis by inhibiting tubulin polymerization and activation of the SAPK/JNK pathway [24], has been shown to arrest growth of mouse spleen and lymphocytic leukaemia cells [44]. Strong telomerase inhibition had been observed in our previous screening studies in human breast cancer and hepatoma cells [27,28]. The telomerase inhibition and senescence induction by chelidonine correlated with down-regulation of the hTERT gene [27,28], while no considerable interactions with telomere sequences were detected in thermal FRET experiments [29]. The catalytic subunit of telomerase has a very low copy number and is regulated in a very complicated manner [45]. Alternative splicing, which plays a critical role in its regulation [34], may shift hTERT transcripts to non-enzyme coding isoforms and suppress telomere elongation in doubling cells [46].

Although we could not see any splice shift with low concentrations of chelidonine, at considerably high concentrations only the hTERT mRNA population shifted towards shorter non-enzyme coding transcripts. This may provide another mechanism for its multiple anti-cancer effects. However, telomere shortening which happened by sequential treatments with very low concentrations, looks to be independent of changes in splicing process. Previous *in vitro* studies showed that chelidonine was not able to any considerable interaction with neither G-quadruplex DNA nor telomerase [37], therefore its strong suppressive effects on telomere length and telomerase is probably through some indirect way.

A similar response to very low concentrations of chelidonine has already been reported in HepG2 cells; HepG2 cells treated sequentially with chelidonine showed reduced cell growth which was arrested after a few repeated treatments [28]. Considering the non-toxic effects of chelidonine in normal cells [47–48], this study may suggest the potential value of this natural product in cancer control and drug design.

## Conclusions

In conclusion, chelidonine, although it does not strongly interact with telomeric G-rich sequences, intensely suppresses telomerase activity leading to progressive telomere shortening in the treated cells. Aside from the hTERT down-regulation, the stronger and stable effects of higher doses of chelidonine may be related to their robust effect on the splicing pattern of the hTERT transcript.
